# The Role of PARP Inhibitors in the Treatment of Gynecologic Malignancies

**DOI:** 10.3389/fonc.2013.00237

**Published:** 2013-10-01

**Authors:** Raquel E. Reinbolt, John L. Hays

**Affiliations:** ^1^Division of Medical Oncology, Department of Internal Medicine, The James Comprehensive Cancer Center, The Ohio State University Wexner Medical Center, Columbus, OH, USA

**Keywords:** ovarian cancer, cervical cancer, endometrial cancer, PARP inhibitor, olaparib, veliparib, rucaparib, niraparib

## Abstract

Gynecologic malignancies annually account for over 91,000 new cancer cases and approximately 28,000 deaths in the United States. Although there have been advancements in cytotoxic chemotherapies, there has not been significant improvement in overall survival in these patients. While targeted therapies have shown some benefit in many solid tumors, further development of these agents is needed for the treatment of gynecologic malignancies. Poly(ADP-ribose) polymerase (PARP) catalyzes the polyADP-ribosylation of proteins involved in DNA repair. Inhibitors of PARP were originally developed for cancers with homologous recombination deficiencies, such as those harboring mutations in *BRCA1* or *BRCA2* genes. However, pre-clinical research and clinical trials have suggested that the activity of PARP inhibitors is not limited to those with *BRCA* mutations. PARP inhibitors may have activity in cancers deficient in other DNA repair genes, signaling pathways that mitigate DNA repair, or in combination with DNA-damaging agents independent of DNA repair dysfunction. Currently there are seven different PARP inhibitors in clinical development for cancer. While there has been promising clinical activity for some of these agents, there are still significant unanswered questions regarding their use. Going forward, specific questions that must be answered include timing of therapy, use in combination with cytotoxic agents or as single-agent maintenance therapy, and whether there is a predictive biomarker that can be used with PARP inhibition. Even with large strides in the treatment of many gynecologic malignancies in recent years, it is imperative that we develop newer agents and methods to identify patients that may benefit from these compounds. The focus of this review will be on pre-clinical data, current clinical trials, and the future of PARP inhibitors in the treatment of ovarian, endometrial, and cervical cancer.

## Introduction

Gynecologic malignancies annually account for over 91,000 new cancer cases and approximately 28,000 deaths in the United States (1). Effective screening for cervical cancer is available in many parts of the world, but there is no effective screening for endometrial or ovarian cancer. Many women with ovarian cancer, therefore, present with advanced stage disease for which cure is rare. Endometrial cancer is more commonly diagnosed early on, as patients are often symptomatic with postmenopausal bleeding. While there have been advancements in the development and administration of cytotoxic chemotherapies, there has not been significant improvement in overall survival in these patients. It is imperative that novel and effective treatment strategies are developed. Although targeted therapies have shown occasional benefit in some solid tumors, these agents have been largely ineffective for the treatment of gynecologic malignancies.

One area of recent interest in targeted therapies for many cancers has been the development of poly(ADP-ribose) polymerase (PARP) inhibitors. PARP catalyzes the polyADP-ribosylation of proteins involved in DNA repair. Inhibitors of PARP were shown to be highly selective for cancer cells that harbor homologous recombination (HR) deficiencies, such as those harboring mutations in *BRCA1* or *BRCA2* genes (2). PARP inhibitors cause an increase in single strand breaks (SSBs) in DNA that, if left unrepaired, will lead to double strand breaks (DSBs) when encountered by replication forks (3, 4). In the laboratory, HR-deficient cells are unable to maintain genomic integrity in the presence of a large number of DNA DSBs and are, therefore, exquisitely sensitive to PARP inhibition. This synthetic lethal interaction between PARP and BRCA has been proposed as a potential explanation for the sensitivity of *BRCA* mutation cell lines to PARP inhibition. Pre-clinical research and clinical trials, however, have suggested that the activity of PARP inhibitors is not limited to those with *BRCA* mutations. PARP inhibitors may demonstrate synthetic lethality in cancers deficient in other proteins that mitigate DNA repair (5). McCabe et al. examined the effects of PARP inhibition on various cell lines deficient in RAD51, Fanconi anemia complementation group (FANC), and Nijmegen breakage syndrome 1 (NBS1), amongst other proteins involved in HR, and found that mutations of these individual proteins induced sensitivity to PARP (6). These findings suggest that the notion of synthetic lethality may be more broadly applied to cancers with an impaired HR pathway, not just those with *BRCA* mutations. This concept is frequently referred to as “BRCAness” or “BRCA-like” (7). The inhibition of SSB repair by PARP inhibition may also be sufficient to enhance the anti-cancer activity in combination with DNA-damaging agents independent of dysfunction in DNA repair pathways (8).

The combination of phosphatase and tensin homolog (PTEN)-deficient cells and PARP inhibition is another area of potential synergistic activity. *PTEN* encodes for a phosphatase that negatively regulates the phosphatidylinositide 3-kinase (PI3K)/AKT/mTOR pathway, which is important for cell proliferation and survival (9, 10) and also plays a poorly understood role in the expression of the DNA repair protein RAD51 and in the functionality of HR. Both *in vitro* and *in vivo* studies have demonstrated sensitivity of PTEN-deficient cells to PARP inhibitors (11–13). Thus, PARP inhibition may benefit patients with malignancies in which there is decreased PTEN expression, such as endometrial cancer, glioblastoma, malignant melanoma, prostate, breast, lung, and colorectal cancers (11).

Currently, there are multiple PARP inhibitors in clinical development for cancer. While there has been promising clinical activity for some of these agents, there are still significant unanswered questions regarding their use. Going forward, specific questions that must be answered include: timing of therapy, use in combination with cytotoxic agents or as a single-agent, maintenance therapy, and the existence of predictive biomarker(s) that can be used with PARP inhibition. Even with large strides in the treatment of many gynecologic malignancies in recent years, it is imperative that we develop newer agents and methods to identify patients that may benefit from these compounds.

## Poly(ADP-Ribose) Polymerase

**Figure 1:**
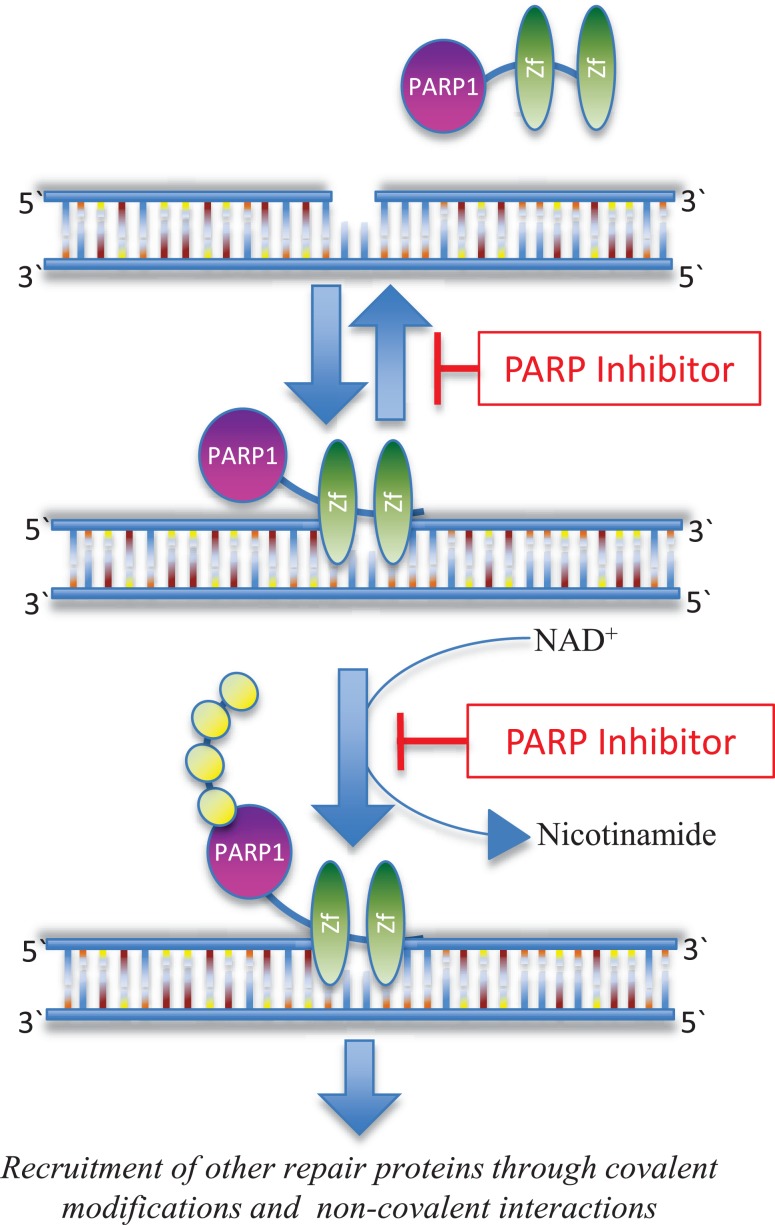
**Inhibition of PARP function**. PARP1 recognizes and binds to sites of DNA damage through its Zn-finger domains (Zf). PARP inhibitors can trap PARP1 on damaged DNA in a complex that is possibly more toxic than unrepaired single strand DNA breaks (28). PARP inhibitors also block the enzymatic activity of the enzyme thereby inhibiting poly(ADP-ribosyl)ation, which in turn blocks recruitment of downstream repair proteins (114).

Base excision repair (BER) is one of multiple critical pathways that maintain genome integrity in all cells, specifically in the recognition and repair of SSBs (14, 15). PARP is a family of 17 proteins that play an important role in DNA repair pathways. The most well studied member of the family, PARP1, is critical in the BER pathway for DNA SSBs. It detects and binds single strand DNA damage sites through its zinc finger domains, next attaching poly(ADP) ribose (PAR) moieties on itself and other proteins that have been recruited to the damage site (Figure 1). If there is excessive DNA damage, such as is seen with ischemia, PARP1 becomes hyperactivated. This heightened activity results in high levels of PAR and the depletion of nicotinamide adenine dinucleotide (NAD^+^) and adenosine triphosphate (ATP) (16), and ultimately, cell death termed parthanatos (17). PARP is also involved in the repair of DSBs (18) and the recruitment of additional repair proteins like ataxia telangiectasia-mutated (ATM) and mitotic recombination 11 (MRE11), both of which are integral to the HR process (19, 20).

PARP1 was first reported in 1963 (21), but its anti-cancer utility was not fully realized until 1980. At that time, Durkacz et al. demonstrated that early-generation PARP inhibitors not only hindered DNA repair, but also enhanced the cytotoxic effects of DNA methylating agents in murine leukemia (22). Kupper et al. demonstrated the enhancement of the cytotoxic effects of gamma-irradiation after reduction of active PARP through overexpression of a dominant negative mutant of PARP that recognizes and binds damaged DNA, but does not possess the catalytic activity of the enzyme (23). More recently, PARP moved into the spotlight with the discovery that PARP inhibition in both cancer cell lines (2, 24) and human tumors (25) lacking BRCA1 or BRCA2 is selectively cytotoxic compared to non-mutation containing tumors. One rationale for this efficacy is a principle termed synthetic lethality, a condition by which deletion or inactivation of only one of two genes (either *BRCA* or *PARP*) would not cause cell death, but deletion or inactivation of two genes in combination (both *BRCA* and *PARP*) is lethal. If PARP1 and PARP2 are inhibited, SSBs typically repaired by BER remain unresolved and when encountered by a replication fork, lead to the accumulation of DSBs (26). BRCA1- or BRCA2-deficient cells lack the ability to effectively complete HR and repair DNA DSBs. This double hit by impairment of both BRCA and PARP functionality ultimately results in genomic instability and cell death. Conferring a potential therapeutic benefit, cell death appears to be limited to homozygous target tissues (i.e., tumor), since most *BRCA* patients carry only one copy of the wild-type *BRCA* gene and there is no apparent effect on cells heterozygous for *BRCA* mutations (2). These observations have been exploited in the treatment of cancers associated with *BRCA* mutations, such as hereditary breast and ovarian cancer (HBOC), and even endometrial cancers (27).

Recently, Murai et al. suggest that the action of PARP inhibition is not only a function of how well the inhibitors disrupt the enzymatic activity, but that certain inhibitors also trap PARP1 on damaged DNA, thereby blocking repair (28). Interestingly, these studies showed that the potency in trapping PARP1 varied among agents, independent of their catalytic inhibitory properties. Clearly, additional investigation is warranted to better understand the intricacies inherent to PARP inhibition pathway and ultimately, advance drug development.

## Hereditary Breast and Ovarian Cancer and BRCA

Hereditary breast and ovarian cancer is typically characterized by the onset of breast cancer at a young age, a strong family history of both breast and ovarian cancer, as well as an autosomal dominant inheritance pattern. Fallopian tube and primary peritoneal cancers also fall into this hereditary spectrum and are included under the ovarian cancer designation. An increased chance of bilateral cancers (e.g., both breasts), the development of both breast and ovarian cancer, and/or an increased incidence of other cancers (pancreas, prostate, etc.) may also be seen in this syndrome. In ovarian cancer, 10% of patients have a genetic predisposition. However, in those patients with a family history of ovarian cancer, the rate of *BRCA1* mutations is 80 and 15% for *BRCA2* mutations (29). More recently with the use of a massively parallel sequencing approach, Walsh et al. identified that closer to 24% of serous ovarian cancer patients have a germline DNA repair defect, over 30% of these were in patients without a family history of breast or ovarian cancer (30). The use of this broader assay is a promising method for detecting germline mutations with greater sensitivity and at decreased cost. Approximately 5–10% of all breast cancers and up to 25–40% of breast cancers in young patients (<35 years old) are hereditary. An estimated 3–8% of all breast cases and 30–40% of familial cases are likely caused by *BRCA1* and *BRCA2* mutations.

Individuals with a *BRCA* mutation have an increased risk of developing ovarian cancer up to 63% by some estimates, and breast cancer by up to 87% (31). Patients with *BRCA1* breast tumors tend to have a higher histologic grade, medullary histopathology, and are more likely than sporadic (non-*BRCA* mutant) tumors to be estrogen receptor negative, progesterone-receptor negative, and HER2/neu overexpression negative or “triple negative” (TNBC) (32). Ovarian cancers associated with *BRCA1* mutation are more often serous adenocarcinomas (90%) compared to women without this mutation (50%) (33–36). Although largely derived from retrospective or indirect data, most studies have not identified a significant survival difference between individuals with *BRCA* mutation-associated breast cancer versus controls (37–44). However, patients with high-grade serous ovarian carcinoma associated with a *BRCA* mutation tend to have a better prognosis than sporadic cases (45, 46). This improved prognosis may be related to *BRCA*-mutated cells’ impaired DNA repair mechanism, lending these lesions greater sensitivity to cytotoxic chemotherapy, especially with platinum-based agents (47, 48). Based on the high selective lethality of *BRCA*-mutated cancer cells to PARP inhibitors, multiple studies have been undertaken to establish efficacy in gynecologic malignancies.

## The Role of PARP Inhibitors in Ovarian Cancer

Although it ranks as the ninth most common cancer among women, excluding non-melanoma skin cancers, ovarian cancer is the fifth most deadly cancer in females and accounts for more deaths than any other cancer of the female reproductive tract in the United States (1). Since the symptoms of disease are typically non-specific, ovarian cancer is often detected in advanced stages when the chance of cure is low. Given its insidious nature and the lethality of the disease, novel therapies are needed to improve overall survival in ovarian cancer patients.

In *BRCA* mutation-associated ovarian cancers, multiple investigations have been completed or are presently underway to establish the clinical activity of PARP inhibition in these mutational carriers. Sixty patients with refractory solid tumors were enrolled in a phase I trial of the PARP inhibitor olaparib (KU-0059436/AZD2281); the study was enriched for patients with *BRCA* mutations (25). In addition to establishing the maximum tolerated dose (MTD) of olaparib at 400 mg bid and observing only minimal adverse effects (primarily fatigue and gastrointestinal), it was noted that only *BRCA* mutation carriers had a significant objective tumor response. Out of 19 patients, 9 had a partial response (PR) (47%) and remarkably, 8 of which were ovarian cancer patients. Twelve of these patients (63%) had either radiological or tumor-marker responses or stable disease for ≥4 months. In an expanded cohort of the same trial, 50 patients with *BRCA1*/2 mutation-associated ovarian, primary peritoneal, and fallopian tube cancers were found to have a clinical benefit rate of 46%, including 40% that experienced a Response Evaluation Criteria in Solid Tumors (RECIST) radiologic or CA125 response (49). The median duration of response was 28 weeks. Another key finding was the overall clinical benefit rate was correlated with platinum sensitivity. Platinum-resistant and refractory patients had a 46 and 23% respective benefit rate versus 69% in the platinum-sensitive population (*P* = 0.038). The study also reported statistically significant associations between the overall platinum-free interval and antitumor response, as well as between platinum sensitivity and the maximum percentage change from radiologic baseline tumor size and from baseline CA125 after olaparib treatment.

In a phase 2 international, multicenter fashion, two sequential cohorts of women with confirmed *BRCA1* or *2* mutations and recurrent disease were given either olaparib at 400 mg twice daily (*n* = 33) or 100 mg twice daily (*n* = 24) (50). The primary efficacy endpoint was objective response rate (ORR). In the 400 mg twice-daily cohort, ORR was 11 of 33 patients (33%; 95% CI 20–51); in the 100 mg twice daily cohort, ORR was 3 of 24 patients (13%; 95% CI 4–31). The most common toxicities experienced included nausea, anemia, and fatigue and were mild in the majority of cases. This phase 2 study provided positive proof of concept for the efficacy and tolerability of olaparib in advanced *BRCA*-mutated ovarian cancer.

Stemming from these initial reports, Kaye et al. designed a phase II, open-label, randomized, international study to assess the safety and efficacy of different doses (200 or 400 mg) of olaparib given twice daily versus intravenous liposomal doxorubicin given monthly in patients with *BRCA*-related ovarian cancer who had failed prior platinum-based chemotherapy (51). A statistically significant higher combined RECIST and CA125 rate of response for olaparib 400 mg twice daily compared to liposomal doxorubicin was noted. It did not find a significant difference in progression free survival (PFS) between the groups, with a reported median PFS of 7.1 months for liposomal doxorubicin, 6.5 months for the 200 mg olaparib cohort, and 8.8 months for the 400 mg olaparib cohort. There were roughly twice as many ≥grade 3 toxicities seen with liposomal doxorubicin compared to the PARP inhibitor. While this study did not show a statistically significant improvement in PFS between olaparib and liposomal doxorubicin, there was a much greater PFS with liposomal doxorubicin (7.1 months) than had been reported in historical data. Gordon et al. demonstrated PFS was only 4 months for liposomal doxorubicin compared to topotecan in a phase III randomized study of recurrent ovarian cancer (52). A recently reported phase III trial by Colombo et al. also demonstrated a similar PFS (3.7 months) for liposomal doxorubicin (53). Although the ability to draw comparisons between studies is limited, Kaye et al. reported PFS with liposomal doxorubicin is still within the 95% CI of historical controls, which suggests that this difference may simply reflect random variation within the population (54).

In addition to their use in *BRCA* mutation-associated ovarian cancer, PARP inhibitors are also being investigated in non-mutation carrier (or *BRCA* wild-type) ovarian cancers. Using PARP inhibitors in such a scenario is based on the idea that there is a HR DNA repair defect, but no germline *BRCA1/2* mutation in up to 50% of ovarian cancers (7, 11, 46, 55). Several studies have exploited this concept. Gelmon et al. conducted a phase II trial with high-grade serous/undifferentiated ovarian cancer with unknown *BRCA* status or *BRCA*-negative disease (56) and an additional reference group with known germline *BRCA* mutations. Patients were treated with olaparib 400 mg twice daily. The ORR in *BRCA*-mutants (*n* = 17) was 41% (95% CI 22–64) with median PFS of 221 days (95% CI 106–383), while *BRCA* mutation negative patients had an ORR of 24% (*n* = 46; 95% CI 14–38) and PFS of 192 days (95% CI 109–267). In a *post hoc* exploratory analysis, the ORR in patients with platinum-sensitive ovarian cancer was 50% (10 of 20) in the *BRCA*-negative cohort and 60% (3 of 5) in the *BRCA*-mutant cohort. In platinum-resistant ovarian cancers, 33 and 4% of patients with *BRCA* mutation positive and *BRCA*-mutant negative status respectively had responses. Observed toxicities were similar to those described in previous studies. This trial’s findings were noteworthy, as they solidified the clinical utility of PARP inhibition in sporadic ovarian cancer. Further, these results suggest that platinum sensitivity may be used as a surrogate marker for HR deficiency. Results of a phase I study of niraparib (MK4827), an oral PARP inhibitor shown to induce selective lethality in HR repair deficient tumors with *BRCA* loss or non-*BRCA* HR defects (57), was given to a small cohort of patients enriched for *BRCA*-deficient and sporadic cancers associated with HR repair defects (58). Thirty-nine patients were treated at 7 successive dose levels; 11 of these patients were *BRCA* mutation carriers. Although results are only available in abstract form, the study reported that three patients with serous ovarian cancer had prolonged RECIST PR (one sporadic platinum-sensitive, two *BRCA*-deficient ovarian cancers). Disease stabilization was observed for >44 weeks in the sporadic serous ovarian cancer patient and for >16 weeks in the two patients with *BRCA*-deficient disease. In another phase II study with the PARP inhibitor rucaparib (AG-014699/PF-0136738), 41 patients with either breast (17) or ovarian (24) cancer and known *BRCA* deficiencies were given rucaparib as monotherapy and followed for ORR (59). Preliminary findings included a clinical benefit rate of 32%, but an ORR of 5% (2/38). However, 26% (10/38) achieved stable disease for ≥4 months and three patients remained on study for >54 weeks. The final results from these two ongoing studies are anxiously awaited.

Another larger, randomized, double-blind, placebo-controlled, phase II trial evaluated maintenance treatment with olaparib in patients with platinum-sensitive, relapsed, high-grade serous ovarian cancer (60). Included patients had received ≥2 platinum-based regimens and were required to have had a partial or complete response to their most recent platinum-based therapy. Two-hundred and sixty five patients were randomized to receive olaparib at 400 mg twice daily or placebo (136 olaparib arm, 129 placebo). *BRCA* mutational status was similar between the two groups. PFS was significantly longer in the olaparib arm than placebo (8.4 versus 4.8 months); however, there was no difference in overall survival at the first interim analysis. Interestingly, subgroup analysis revealed that regardless of *BRCA* mutational status, the olaparib cohort had a decreased risk for progression. Toxicities were overall mild in the olaparib group; most adverse events were grade 1 or 2 and typically included nausea, fatigue, vomiting, and anemia. These findings again support the argument that platinum sensitivity is a useful clinical marker for olaparib sensitivity. Further, this investigation recapitulates the role of PARP inhibitors in the ovarian cancer population, regardless of *BRCA* mutational status, and underscores the need for development of relevant biomarkers that predict HR deficiency in the setting of *BRCA* mutations or no known genetic abnormalities. Fortunately, there are multiple ongoing trials investigating the relationship between PARP inhibition and ovarian cancer that will hopefully clarify some of these uncertainties (Table 1).

**Table 1:** **Active clinical trials investigating PARP inhibitors in gynecologic malignancies**.

Agent	Clinical trial identifier^E^	Trial description	Phase	Combination or monotherapy
Olaparib^A^	NCT01237067	Olaparib in combination with carboplatin for refractory/recurrent women’s cancers	1	Combination
	NCT01116648	Olaparib in combination with cediranib for recurrent ovarian or TNBC	1/2	Combination
	NCT01445418	Olaparib with carboplatin to treat breast and ovarian cancer	1	Combination
	NCT01623349	Olaparib with BKM120 in recurrent TNBC or high-grade serous ovarian cancer	1	Combination
	NCT01650376	Olaparib with carboplatin and paclitaxel in relapsed ovarian cancer	1b	Combination
	NCT00782574	Olaparib with cisplatin in advanced solid tumors	1	Combination
	NCT00628251	Olaparib versus doxorubicin in advanced BRCA1/2 ovarian cancer patients who have failed previous platinum-therapy	2	Monotherapy
	NCT01844986	Olaparib in BRCA-mutated ovarian cancer patients following first line platinum-based chemotherapy	3	Monotherapy
	NCT01078662	Olaparib in advanced cancers with a confirmed BRCA1/2 mutation	2	Monotherapy
	NCT01874353	Olaparib in BCRA mutated ovarian cancer patients after complete or partial response to platinum chemotherapy	3	Monotherapy
	NCT00516373	Olaparib in ovarian cancer	1	Monotherapy
Veliparib^B^	NCT00989651; GOG-9923	Veliparib in combination with carboplatin, paclitaxel, bevacizumab for newly diagnosed ovarian, fallopian tube, or primary peritoneal cancer	1	Combination
	NCT01306032	Veliparib with cyclophosphamide in refractory BRCA-positive ovarian, primary peritoneal, ovarian high-grade serous carcinoma, fallopian tube cancer, TNBC, low-grade non-Hodgkin’s lymphoma	2	Combination
	NCT01459380; GOG 9927	Veliparib in combination with doxorubicin, carboplatin, and bevacizumab	1	Combination
	NCT01281852; GOG-0076HH	Veliparib with cisplatin and paclitaxel in patients with advanced, persistent, or recurrent cervical cancer	1/2	Combination
	NCT01145430	Veliparib and doxorubicin for recurrent ovarian, fallopian tube, and primary peritoneal cancers or metastatic breast cancer	1	Combination
	NCT01266447; GOG 127-W	Veliparib, topotecan, and filgrastim or pegfilgrastim in patients with persistent/recurrent cervical cancer	2	Combination
	NCT01690598	Veliparib with topotecan in patients with platinum-resistant or partially platinum-sensitive relapse of epithelial ovarian cancer with negative or unknown BRCA status	1/2	Combination
	NCT01012817	Veliparib with topotecan in relapsed/refractory or primary peritoneal cancer after prior first line platinum-therapy	2	Combination
	NCT01113957	Veliparib with temozolomide versus doxorubicin alone in ovarian cancer	2	Combination
	NCT01749397	Veliparib and floxuridine in metastatic epithelial ovarian, primary peritoneal, or fallopian tube cancer	1	Combination
	NCT01540565; GOG-0280	Veliparib in persistent or recurrent epithelial ovarian, fallopian tube, or primary peritoneal cancer patients with a BRCA2 mutation	2	Monotherapy
	NCT00892736	Veliparib monotherapy for patients with BRCA1/2 -mutated cancer, including platinum-refractory ovarian, fallopian tube, or primary peritoneal cancer; or basal-like breast cancer	1	Monotherapy
	NCT01472783	Veliparib for patients with BRCA mutation and platinum-resistant or partially sensitive relapse of epithelial ovarian cancer	1/2	Monotherapy
BMN 673	NCT01286987	BMN 673 in advanced or recurrent solid tumors, including epithelial and ovarian cancers	1	Monotherapy
Niraparib^C^	NCT01847274	Niraparib versus placebo in platinum-sensitive ovarian cancer	3	Monotherapy
Rucaparib^D^	NCT01009190	Rucaparib with carboplatin in advanced solid tumors	1	Combination
	NCT01482715	Rucaparib in patients with BRCA mutation breast or ovarian cancer, or other solid tumor	1/2	Monotherapy
	NCT00664781	Rucaparib in metastatic breast cancer or ovarian cancer	2	Monotherapy

^A^ Olaparib, also known as AZD2281.

^B^ Veliparib, also known as ABT-888.

^C^ Niraparib, also known as MK-4827.

^D^ Rucaparib, also known as AG-014699; PF-01367338.

^E^ All clinical trials are found at www.clinicaltrials.gov and listed according to their NCT identifier. Last accessed 2013 June 19.

## PARP Inhibitors in Endometrial Cancer

Endometrial cancer is the fourth most common cancer in women and the most commonly diagnosed gynecologic malignancy. An estimated 90% of the cases are sporadic and 10% have a genetic origin. Endometrioid adenocarcinoma and serous carcinoma are the most prevalent histological types, while endometrial clear cell and mucinous carcinomas only account for approximately 5% of all cases (61). Since many patients are symptomatic early in their disease course, the majority of endometrial cancers (approximately 75%) are detected in the initial stages when the disease remains confined to the uterus (61). However, a significant amount of women still experience advanced disease, for which systemic treatment options are limited, toxicities high, and responses often short-lived (62, 63). There is a pressing need for targeted therapies that will yield a greater efficacy and be better tolerated.

A variety of different molecular defects linked to the development of endometrial cancer are described. In endometrioid endometrial carcinoma (EEC), also known as type I endometrial cancer, microsatellite instability (MSI) and mutations in the *PTEN*, *K-ras*, *PIK3CA*, and β*-catenin* genes are reported (64). As previously discussed, *PTEN* is a tumor suppressor gene that is involved in DNA repair mechanisms, as well as in the inhibition of the PI3K/AKT/mTOR pathway; *PTEN*-deficient cells are sensitive to PARP inhibitors (11–13). Rare syndromes collectively known as the PTEN hamartoma tumor syndromes (PHTS) are linked to germline mutations in *PTEN* (65, 66). Outside of PHTS, *PTEN* is altered in up to 83% of endometrioid carcinomas versus only 10% in serous and clear cell cancers (67–71). Dedes et al. demonstrated that *PTEN*-deficient EEC cells had a greater sensitivity to PARP inhibition than wild-type EEC *PTEN* cell lines (12). Given the heightened prevalence of *PTEN* deficiency in EEC superimposed on these laboratory studies demonstrating sensitivity to PARP inhibition, clinical studies are now in progress. A case report describing a 58-year-old female with metastatic endometrioid endometrial adenocarcinoma who had previously demonstrated exquisite sensitivity to platinum-containing regimens, was given olaparib as part of a phase I trial (72, 73). Prior to trial participation, brain metastases were found. However, after 10 weeks on trial, the patient had a significant reduction in the size of the brain metastases without other intervention and also reported improvement in tumor-related symptoms. Unfortunately, the patient had objective disease progression after 8 months on olaparib therapy. Her tumor was biopsied and verified to be negative for *BRCA* mutation, but positive for loss of *PTEN*. Although only an isolated report, this case study coupled with compelling pre-clinical data, provides a strong rationale for larger clinical trials. A phase 2, randomized, placebo-controlled trial comparing olaparib versus best supportive care or progesterone in advanced endometrial cancer was planned, but unfortunately, was unable to be opened. In addition to EEC, serous endometrial cancers appear to have a similar genetic background to serous ovarian carcinoma, including hallmarks of deficiency in DNA repair as well as frequent mutations in *TP53*, *PIK3CA*, *K-RAS*, and *ERBB2* (74). These tumors may prove to be another rational target for PARP inhibition.

## PARP Inhibitors in Cervical Cancer

As the third most common cancer worldwide, cervical cancer has an annual incidence of 530,000 cases, with 250,000 deaths expected (75). It is the second leading cause of death in women from the ages of 20–39 (76). Fortunately, the incidence of this cancer in most developed countries has decreased by 70% over the past 50 years due to improved screening methods with cervical cytology (77). More recently, HPV vaccination has aided in the detection and subsequent prevention of high-risk HPV subtypes, which are the culprit for most cervical cancers (78–82). For advanced disease, chemotherapy remains the standard of care. Similar to the experience in endometrial cancer, such therapy typically does not yield durable responses or cure (83).

The use of PARP inhibitors in cervical cancer has only recently been explored in the pre-clinical arena. Along with non-small cell lung cancer, mesothelioma, and ovarian cancer cell lines, Michels et al. created cervical cancer (HeLa) cell lines resistant to cisplatin (84). Upon further study, these lines were found to have high levels of PAR and PARP1, with PARP1 constitutively hyperactivated. Exposure of the cells to pharmacologic PARP inhibition resulted in cell death. Hence, this work hints at another role for PARP inhibition, in the treatment of cisplatin-resistant cervical cancers. Interestingly, this group also observed that elevated levels of PAR identified in PARP1-overexpressing tumor cells and xenografts predicted response to PARP inhibition *in vitro* and *in vivo* more accurately than PARP1 expression itself, suggesting PAR may be a reasonable biomarker of response to PARP inhibitor therapy in cervical cancer. A phase I trial is presently enrolling patients with cervical cancer along with other gynecological malignancies to investigate the combination of olaparib with carboplatin in refractory or recurrent disease (NCT01237067; see Table 1). Another phase 1/2 trial is investigating the use of veliparib with cisplatin and paclitaxel in advanced, persistent, or recurrent cervical cancer (NCT01281852; Table 1). Additional pre-clinical and clinical investigation will hopefully reveal even more promising applications for PARP inhibition in cervical cancer.

## Future Directions

Poly(ADP-ribose) polymerase inhibitors are an exciting new class of agents that have already demonstrated promising pre-clinical and clinical activity in a variety of malignancies. Nevertheless, the full potential of PARP inhibition in cancer has not yet been realized. In addition to single-agent use, PARP inhibitors have been studied in combination with a number of different chemotherapies, anti-angiogenic agents, as well as with ionizing radiation. Other areas of active investigation include the development of markers that will predict clinical benefit from PARP inhibition, as well as the identification of resistance mechanisms to PARP inhibitor therapy.

Chemotherapies known to induce DNA strand breaks, especially SSBs, are of particular interest for combination studies. In the case of methylating agents, activation of BER elicits therapy resistance (85). A large body of pre-clinical *in vivo* and *in vitro* studies demonstrates the addition of a PARP inhibitor may sensitize cells to DNA-damaging agents and further delay the development of treatment resistance (8, 85–93). These studies were conducted with a wide variety of chemotherapeutic agents, including topoisomerase I inhibitors, platinum agents, as well as DNA alkylating agents. Human trials combining PARP inhibitors and chemotherapy agents for sporadic and *BRCA*-associated gynecologic malignancies are underway, but few have reached maturity (NCT01445418, NCT01237067; see Table 1). Promising data has come from Oza et al., who conducted a multicenter phase II study that compared the efficacy of olaparib plus paclitaxel/carboplatin followed by olaparib maintenance therapy versus paclitaxel/carboplatin alone with no further therapy in patients with platinum-sensitive recurrent serous ovarian cancer (94). Importantly, the *BRCA* status was unknown for the majority of the patients. In arm A, patients received six, 21-day cycles of olaparib (200 mg twice daily) with paclitaxel (175 mg/m^2^ IV, day 1) and carboplatin (AUC 4 IV, day 1), followed by olaparib maintenance therapy at a dose of 400 mg twice daily in a continuous fashion versus in arm B, the standard dose of carboplatin (AUC 6 IV, day 1) and paclitaxel (175 mg/m^2^ IV, day 1) without the PARP inhibitor. Patients receiving olaparib had a significant improvement in PFS versus chemotherapy alone. OS data was felt to be immature, but preliminarily showed similar results between the two arms (64 versus 58%). In the combination phase, both arms had generally similar toxicity profiles, with nausea, fatigue, and alopecia the most common adverse events experienced. During the maintenance phase (olaparib monotherapy versus no further therapy), side effects were consistent with the known monotherapy side effect profile of PARP inhibitors. In a smaller phase I dose escalation trial, olaparib was added to carboplatin in *BRCA1*/2 mutational carriers with breast or ovarian cancer (95). Therapy was administered in a 3 × 3 dose escalation fashion: oral olaparib at 100 or 200 mg every 12 h [dose level (DL) 1/2] with IV carboplatin AUC 3 on day 8 then every 21 days; DL6–9 gave olaparib days 1–7 at 200 then 400 mg every 12 h, with carboplatin AUC 3 on day 2 then escalation to AUC 5 (no DL3–5). From the preliminary results, bone marrow suppression was the observed dose limiting toxicity. Of the 23 evaluable ovarian cancer patients, PR was seen in 8/23, disease stabilization occurred in 11/23. Overall, the ovarian cancer cohort had a clinical benefit of 83%. Clearly, the results of these studies are intriguing; data from similar combination trials is eagerly anticipated.

In addition to chemotherapeutic agents, PARP inhibitors are also being combined with anti-angiogenic agents. The rationale behind this combination is based on the observation that vascular endothelial growth factor receptor (VEGFR) inhibition may lead to increased DNA damage through downregulation of DNA repair proteins, including ERCC1 and XRCC1 (96, 97). Stemming from pre-clinical data supporting the relationship between PARP inhibition and the VEGF pathway (98–100), several phase I studies are presently underway. The phase 1 study of ABT-888 (veliparib) in combination with carboplatin, paclitaxel, and bevacizumab as first-line treatment for stage II-IV ovarian cancer is actively enrolling patients (NCT00989651; Table 1). Another phase I trial of olaparib in combination with cediranib, a VEGFR inhibitor, is also open to recurrent ovarian or TNBC patients (NCT01116648; Table 1). Trial investigators are exploring the toxicities and recommended phase 2 dosing of the dual therapy. From a preliminary report, myelosuppression was dose limiting at the highest dose level (cediranib 30 mg daily/olaparib 400 mg twice daily) (101). Although unconfirmed, the study also notes a 56% response rate in enrolled ovarian cancer patients. These results are encouraging; additional efficacy data will be forthcoming (Table 1).

Due to PARP’s ability to inhibit multiple processes related to DNA repair, combining PARP inhibition with ionizing radiation is a logical combination. Pre-clinical studies confirm that PARP inhibition acts to sensitize malignant cells to radiation (88, 102). Several laboratories have also shown that PARP 1 knockout mice have an enhanced sensitivity to gamma-radiation (103, 104). In mouse colon cancer xenografts, veliparib coupled with irradiation resulted in prolonged survival from 23 to 36 days, and in one mouse, a complete response (8). At the present time, there are no active clinical trials investigating the combination of radiation therapy with PARP inhibition in gynecologic malignancies. However, there are active trials investigating this dual therapy in other diseases like breast cancer (NCT01477489) (105) and glioblastoma multiforme (NCT00687765) (106). Enrollment of gynecologic malignancy patients into similar trials is important since radiation plays a significant role in the treatment of cervical and endometrial cancer.

As evidenced by the discussed clinical data, many patients benefit from PARP inhibitor therapy, though the degree of response varies and sometimes there is no observed clinical benefit. A predictive marker that not only evaluates the drug’s pharmacodynamic effects, but can also identify who might benefit from therapy may help guide treatment decisions. Several attempts have been made to meet this objective. Duan et al. described a triple stain immunofluorescence assay looking at FANCD2, DAPI, and Ki67 as a means for measuring the functional competency of the Fanconi anemia pathway in proliferating cells in formalin fixed tumor tissue from patient biopsies across multiple tumor types (5). This stain is now being tested in a prospective fashion to select patients for a phase 1 clinical trial using veliparib alone or in combination with mitomycin-C (NCT01017640). The use of massively parallel sequencing analysis (e.g., BROCA) in a prospectively designed trial should also be investigated as this may capture a larger percentage of patients likely to be sensitive to PARP inhibition compared to relying on BRCA1/2 mutational analysis alone (30). Mukhopadhyay et al. developed a method of measuring HR function by quantifying RAD51 foci via immunofluorescence-based assays of ascitic fluid (107). They subsequently correlated *in vitro* cytotoxicity of the PARP inhibitor rucaparib with the HR status from these culture results. They correlated their *in vitro* results to patients whom were treated with platinum-based chemotherapy; tumor progression and OS were prospectively compared between HR-competent versus HR-deficient patients (108). Interestingly, patients who were HR-deficient, as established by assay analysis, had lower rates of tumor progression at 6 months and a higher median survival. From these results, the authors suggest that the RAD51 assay successfully identified those patients with HR deficiency and hence, may better predict which patients will have the best response to PARP inhibition. In addition to ascitic fluid, collection of peripheral blood mononuclear cells (PBMCs) as a surrogate tissue to monitor drug actions may be preferable to tumor biopsy collection, as it is less invasive and multiple samples may be longitudinally obtained. In order to better characterize the pharmacodynamic profile of the PARP inhibitor ABT-888, Ji et al. developed an immunoassay for measuring PAR incorporation in both tumor biopsies and PBMCs (109). In this study, considerable inter-individual and inter-sample heterogeneity in PAR levels was observed. Given these findings, it is not surprising that the trial comparing cyclophosphamide with veliparib presented a 50% reduction in PAR levels in 90% of patient PBMCs and 80% reduction in tumor biopsies across all dose levels (110). A larger phase II follow up study with this combination is ongoing (NCT01306032; Table 1). Though limited conclusions may be drawn from this experience, one must consider the possibility that PAR levels did not correlate well with actual PARP inhibitor activity (111). Ongoing genomic microarray analysis of patients involved in trials using olaparib may give useful insight into genetic signatures that may predict response. Regardless, these results underscore the need to identify a validated method of quantifying PARP inhibitor activity that corresponds to actual clinical outcome.

As with the majority of anti-cancer agents, tumors may develop acquired resistance to PARP inhibitor therapy. There are several proposed mechanisms of resistance, and likely many more that have not yet been described. One potential means is the restoration of HR secondary to a gain of function mutation in the *BRCA2* allele via elimination of the c.6174delT mutation (112). Resistance secondary to up regulation of the *ABCB1a/b* gene that encodes for a P-glycoprotein efflux pump is also described with long-term use of the PARP inhibitor olaparib. Reversal of resistance occurred with co-administration of a P-glycoprotein inhibitor (113). These are just two examples of methods of resistance and certainly the success of PARP inhibitor therapy in the future will rely on further analysis of resistance patterns and subsequent therapy modifications.

## Conclusion

Gynecologic malignancies represent a significant challenge in women’s health. When discovered in advanced stages, few successful therapeutic interventions are available to patients. Therefore, the development of novel agents like PARP inhibitors is essential. Already recognized as a promising agent in the treatment of *BRCA*-related malignancy, initial phase I and II studies confirm the activity of PARP inhibitors in ovarian, endometrial, and cervical cancers. As we learn more about these targeted agents through ongoing trials, it will be important to identify which population of patients may benefit the most from PARP inhibitor therapy and in what manner, as monotherapy or in combination. Whether it is in the neoadjuvant, adjuvant, or maintenance setting, the timing of therapy that will procure the greatest clinical benefit is also unknown. Clearly, PARP inhibitors are an exciting new class of targeted agents for the treatment of ovarian, endometrial, and cervical cancers.

## Conflict of Interest Statement

The authors declare that the research was conducted in the absence of any commercial or financial relationships that could be construed as a potential conflict of interest.

## Abbreviations

ATP, adenosine triphosphate; ATM, ataxia telangiectasia-mutated; BER, base excision repair; DL, dose level; DSB, double strand breaks; EEC, endometrioid endometrial carcinoma; FANC, Fanconi anemia complementation group; HBOC, hereditary breast and ovarian cancer; HR, homologous recombination; MRE11, mitotic recombination 11; MSI, microsatellite instability; MTD, maximum tolerated dose; NAD^+^, nicotinamide adenine dinucleotide; NSB1, Nijmegen breakage syndrome; ORR, objective response rate; PAR, poly(ADP) ribose; PARP, poly(ADP-ribose) polymerase; PFS, progression free survival; PI3K, phosphatidylinositide 3-kinase; PHTS, PTEN hamartoma tumor syndromes; PMBCs, peripheral blood mononuclear cells; RECIST, response evaluation criteria in solid tumors; SSBs, single strand breaks; TNBC, triple negative breast cancer; VEGFR, vascular endothelial growth factor receptor.
